# Treatment of the Vomiting of Pregnancy by Mechanical Appliances

**Published:** 1876-02

**Authors:** 


Treatment of the Vomiting of Pregnancy by Mechanical Appliances to the Os Uteri.—Some time since the attention of the profession was called to a method of arresting the sickness of pregnancy by dilating moderately the os uteri. The effect of dilatation was brought to light through its application for the purpose of bringing on abortion. It was discovered that the vomiting ceased under these circumstances, and gestation continued undisturbed. To explain the phenomenon various suggestions were made, one of which was that "dilatation of the neck detaches the membranes over a certain space, and prevents the twitchings or distention of the internal orifice." A French obstetrician alleges that he has obtained the same results from the simple application of a plug or wadding to the vagina, which acts, he supposes, by "preventing the shaking about of the womb." Rather a fanciful explanation, we should say. A more rational supposition will suggest itself to most minds, namely: the principle of counter-irritation—the same principle which enables a blister on the surface of the body, under certain circumstances of nervous excitement and wakefulness, to act as an anodyne and induce sleep.—*Pacific Med. and Surg. Jour.*

